# Smoking accelerates immunosenescence in multiple sclerosis

**DOI:** 10.3389/fimmu.2026.1840928

**Published:** 2026-06-29

**Authors:** Meerah Khan, Ekdanai Uawithya, Joshua S. Mytych, Ismail Muwenda, Megan Reidy, Robert C. Axtell, Yang Mao-Draayer

**Affiliations:** Arthritis and Clinical Immunology Program, Oklahoma Medical Research Foundation, Oklahoma, OK, United States

**Keywords:** cigarette smoking, disease progression, immunosenescence, multiple sclerosis, neuroinflammation, oxidative stress, relapse

## Abstract

Multiple sclerosis (MS) is a chronic immune-mediated disease of the central nervous system characterized by inflammation, neuroaxonal injury, and progressive neurologic disability. Although its etiology is multifactorial, cigarette smoking is among the most consistent modifiable environmental exposures associated with increased MS risk and worse clinical outcomes. Smoking has been linked to greater inflammatory activity, accelerated disability accumulation, and earlier progression, suggesting effects across multiple stages of disease biology. In this review, we examine the hypothesis that smoking accelerates immune aging, or immunosenescence, and thereby contributes to both early inflammatory disease activity and later neurodegenerative progression. We summarize evidence that smoking promotes immune phenotypes resembling physiologic aging, including contraction of naïve T-cell pools and expansion of terminally differentiated, senescent CD8+ populations. We also discuss potential mechanisms underlying these effects, including oxidative stress, cholinergic signaling, and epigenetic remodeling. By integrating epidemiologic, clinical, and mechanistic evidence, we propose that smoking-associated immune aging acts as a biologic modifier of MS, influencing susceptibility, relapse activity, and long-term progression.

## 1 Introduction

Multiple sclerosis (MS) is a chronic immune-mediated disorder of the central nervous system characterized by inflammatory demyelination, neuroaxonal injury, and progressive neurological disability. While the etiology of MS is multifactorial, involving both genetic susceptibility and environmental exposures, cigarette smoking has consistently emerged as one of the most robust and modifiable environmental risk factors. Smoking has been associated with increased MS susceptibility, higher disease activity, accelerated disability accumulation, and earlier conversion to secondary progressive MS across multiple longitudinal cohort studies (1–4).

The biological mechanisms through which smoking influences MS remain incompletely understood. Proposed pathways include enhanced systemic inflammation, oxidative stress, epigenetic modification, and altered immune cell function (5). However, these mechanisms are often considered in isolation, without a unifying framework explaining how smoking contributes to both inflammatory disease activity and later neurodegenerative progression. Notably, many of the immunologic effects attributed to smoking parallel changes observed in immune aging, suggesting that smoking may accelerate age-associated immune dysfunction.

In this review, we examine the evidence linking cigarette smoking to immune aging and discuss how this may influence MS susceptibility, disease activity, and progression. By integrating epidemiologic data, immunologic studies, and mechanistic insights, we propose that smoking acts as an accelerator of immune aging in MS, contributing to earlier immune exhaustion, altered treatment responsiveness, and worse long-term outcomes. Understanding this relationship has important clinical implications, particularly as the MS population ages and cumulative exposure to both disease-modifying therapies and environmental risk factors increases.

Importantly, the clinical effects of smoking in MS are not limited to progression alone. Epidemiologic studies associate smoking with increased risk of MS diagnosis, higher relapse activity, and faster disability accumulation, suggesting that smoking acts across multiple stages of disease biology. Thus, smoking may best be understood as a broad biologic modifier of MS, with stage-specific effects spanning disease initiation, inflammatory activity, and progressive decline. While prior studies have examined smoking as a risk factor in MS and others have described features of immunosenescence, which is the gradual remodeling of the immune system that occurs with aging, in the disease, these domains are often considered separately (6). A key gap in the literature is the lack of an integrated framework that links smoking-associated immune remodeling with immune aging processes across the MS disease continuum. This review addresses this gap by proposing that smoking acts as an accelerator of immunosenescence, thereby providing a unifying mechanism connecting susceptibility, relapse activity, and long-term progression.

## 2 Immunosenescence: definitions and mechanisms

Immunosenescence is characterized by loss of naïve lymphocytes, expansion of highly differentiated or senescent T-cell subsets, and reduced capacity to respond to new antigens (7–10). Rather than simply reflecting immune failure, immunosenescence encompasses coordinated changes across adaptive and innate immunity, including persistent low-grade inflammation and impaired capacity to respond to physiological stressors. These changes are increasingly recognized as contributors to age-related susceptibility to chronic inflammatory and neurodegenerative diseases. Several studies suggest that people with MS exhibit features consistent with premature immune aging, including reduced naïve T-cell populations, altered T-cell homeostasis, and expansion of differentiated immune phenotypes even in younger patients (11–15).

The intersection of smoking, immunosenescence, and MS may be particularly relevant to understanding disease progression. Long-term cohort studies show that, with increasing age and disease duration, MS often transitions from predominantly relapsing inflammatory activity toward a progressive phase characterized by accumulating disability (16, 17). During this phase, relapse frequency often declines, while disability continues to accumulate. Immunosenescence provides a conceptual framework that may help reconcile this paradox, as aging immune systems may exhibit reduced acute inflammatory responses while sustaining chronic, tissue-damaging immune activity (7, 18). Earlier effects on susceptibility and relapse activity likely involve complementary mechanisms such as oxidative stress, epigenetic remodeling, enhanced peripheral immune activation, and altered immune tolerance.

Cigarette smoke exerts broad and multifaceted effects on the immune system that extend beyond generalized inflammation. Exposure to tobacco smoke has been shown to alter both innate and adaptive immune responses, including disruption of T-cell differentiation, promotion of pro-inflammatory cytokine production, and impairment of immune regulatory pathways (19, 20). At the molecular level, smoke-related oxidants and reactive aldehydes induce oxidative stress, DNA damage, and epigenetic modifications, which can collectively reshape immune cell function (21, 22). These effects are accompanied by persistent immune activation and altered signaling networks, contributing to a state of chronic low-grade inflammation. Importantly, many of these changes parallel features observed in immune aging, suggesting a potential mechanistic link between smoking exposure and immunosenescence.

At the molecular level, mechanisms of immunosenescence are the result of genomic instability, accumulation of damage, and epigenetic modifications that trace back to the most foundational units of self - DNA (23). Genomic instability is often due to defects in the DNA damage response (DDR) pathway or DNA replication machinery, and has been implicated as a factor in inflammation-driven response in human aging (24). Epigenomic studies have found that there are also changes in both peripheral and resident MS patient immune cells with age (25, 26). Multi-omic analyses reveal that smoking-induced molecular changes — including gene expression shifts and age-associated DNA methylation patterns — significantly overlap with aging signatures across multiple tissue types, suggesting that smoking may directly accelerate biological aging mechanisms (27).

At the cellular level, a central feature of immunosenescence is the decline in naïve T-cell production as thymic involution progresses with age. Repeated antigen exposure and chronic low-grade inflammation further drive expansion of terminally differentiated immune cells and contribute to a persistent pro-inflammatory environment often termed inflammatory aging (inflammaging). Longitudinal cohort and population-based studies show that elevated inflammatory markers such as IL-6 and CRP predict physical decline and mortality in aging populations, supporting the role of sustained inflammation in immune aging (28–30). Comparable age-related alterations occur within the B-cell compartment, including reductions in naïve B cells, accumulation of more differentiated subsets, and narrowing of antibody diversity, reflecting impaired humoral immune adaptability with aging (31, 32).

Innate immune cells also undergo age-associated functional changes. Microglia and macrophages show altered activation thresholds, impaired debris clearance, and dysregulated cytokine production, changes that become increasingly pronounced in aging brain tissue (33–35). These alterations may promote inefficient repair and gradual neurodegeneration. Transcriptomic analyses of purified human microglia further demonstrate age-associated shifts in inflammatory and homeostatic gene expression programs, supporting the concept that microglial aging contributes to chronic neurodegenerative processes relevant to progressive MS (36, 37). Recent transcriptional profiling studies further demonstrate that human microglia exhibit age-associated immunometabolic and inflammatory pathway alterations, reinforcing the role of microglial aging in neurodegenerative processes relevant to progressive MS (37). The resulting immune profile is paradoxical: acute responses may be attenuated, yet persistent, tissue-damaging inflammatory activity is sustained.

One potential cause of chronic, low-level inflammation may be age-related changes. Both innate and adaptive immune cells can take on a senescence-associated secretory phenotype (SASP), a key feature of aged cells, noted by a modified secretory profile that includes a high level of inflammatory cytokines, growth factors and metalloproteinases including IL-6, TNF, and growth/differentiation factor 15 (GDF15), cytokines increasingly recognized as central mediators of age-related inflammatory disease processes (38–40). SASP not only denotes a damaged cell but also actively participates in modifying both neighboring and distant cells and is implicated in increasing immune surveillance (41, 42). While acutely beneficial, long-term consequences of age-related changes can include defective immune functioning, including promoting tissue 0fibrosis, and modifying immune checkpoint expression, among many other processes (43, 44). Recent studies have implicated SASP and aging with faster disability progression in MS patients, and defective remyelination in murine MS models (45, 46).

In addition to immune and age-related defects, metabolic changes are an alternative cell-based mechanism that is established in MS progression. Metabolic defects typically manifest as mitochondrial and oxidative damage and are defective within MS lesions, plaques, and peripheral components in patients (47–50). Among evidence pointing to oxidative damage, one of the clearest is production of 8-oxo-dG, an oxidative compound synthesized as a result of reactive oxygen species (ROS)-induced damage to DNA, and in particular via inhaling tobacco smoke (51, 52). This compound is elevated in both urine and cerebrospinal fluid of MS patients and is only one of many factors that change with both oxidative damage and smoking in MS (53, 54).

These studies set up a framework that is particularly relevant for understanding changes in MS biology over the lifespan. Early in the disease, peripheral adaptive immune activation drives relapsing inflammatory activity. With advancing age and disease duration, MS increasingly shifts toward a phenotype dominated by compartmentalized CNS inflammation, microglial activation, and neurodegeneration, while overt relapses become less frequent (16, 17, 55, 56). Consistent with this transition, immunophenotyping studies in progressive MS demonstrate reduced naïve T-cell populations and increased effector-memory and differentiated T-cell subsets, supporting the presence of age-like adaptive immune remodeling in later disease stages (57). Immunosenescence offers a plausible biologic explanation for this transition, helping reconcile declining relapse rates with ongoing disability accumulation. Taken together, these insights suggest that factors capable of accelerating or amplifying immunosenescence may meaningfully influence MS susceptibility and progression. (See **Table 1** for key immunosenescence features in MS).

**Table 1:** Immunosenescence features observed in people with multiple sclerosis.

Immunosenescence feature	Description in MS	Implication	Key references
Reduced naïve T-cell pools	MS patient.- including younger adults – show reduced thymic output and fewer naïve CD4*T-cells.	Suggests premature immune aging and early contraction of T-cell diversity.	(12, 13, 15)
Shift toward memory/exhausted T-cells	Expanded effector-memory populations and altered CD8* T-cell differentiation patterns observed in MS.	Reduced immune adaptability and resilience.	(14, 57, 58)
Chronic immune activation	Evidence of ongoing immune activity even during clinically stable phases or despite therapy.	Persistent inflammatory drive contributing to tissue damage.	(56, 59)
Microglial activation	Aging-like microglial activation and compartmentalized CNS inflammation in progressive MS.	Promotes neurodegeneration and progression.	(34, 37, 60)
Systemic “inflammaging”	Elevated circulating inflammatory markers are linked with aging-related decline and mortality.	Provides biologic linkage between aging, inflammation, and worsening outcomes.	(30, 36, 40);

This table summarizes immune-aging characteristics described in MS, including reduced naïve lymphocyte pools, expansion of differentiated/exhausted T-cell subsets, persistent immune activation, and chronic microglial involvement, all of which may contribute to progressive disability.

## 3 Smoking as an accelerator of immunosenescence

Cigarette smoking exerts widespread effects on the immune system that extend beyond classical inflammation and oxidative injury. Increasing evidence indicates that chronic smoking promotes immune changes that closely resemble, and may accelerate immunosenescence. These mechanistic links between smoking-related immune alterations and immunosenescence features are summarized in **Table 2**. These alterations include shifts in lymphocyte composition, cellular aging phenotypes, and dysregulated immune signaling, all of which have potential relevance to MS pathophysiology (69–71).

**Table 2:** Proposed pathway linking smoking, immunosenescence, and multiple sclerosis.

Smoking-related exposure/effect	Immunosenescence feature	Biological implication	Relevance to MS	Key references
Oxidative and DNA-damaging stress	Accelerated immune aging	DNA damage, epigenetic changes, and long-term immune dysfunction	Earlier progression and reduced repair capacity	(5, 27, 61)
Altered T-cell signaling	Dysregulated effector responses	Altered activation thresholds and impaired immune regulation	Ongoing CNS injury and reduced therapeutic efficacy	(62, 63)
Smoking-associated immune remodeling	Expansion of senescent CD8^+^ T cells	Increased dysfunctional cytotoxic T-cell populations	Sustained inflammation and disease progression	(18, 64)
Smoking-associated T-cell shifts	Altered CD4^+^/CD8^+^ balance	Age-like skewing toward memory/effector phenotypes	Shift toward progressive, less inflammatory disease	(65)
Persistent immune remodeling	Long-lasting immune dysfunction	Durable changes in adaptive immunity after exposure	Chronic dysregulation and altered disease trajectory	(66)
Repeated inflammatory activation	Expansion of senescent T cells	Accumulation of low-function, pro-inflammatory cells	Chronic inflammation, microglial activation, neuroaxonal injury	(28, 29, 67, 68)

This table summarizes mechanistic links between smoking-related immune alterations, features of immunosenescence, and potential consequences for MS pathophysiology. Experimental, immunologic, and systems-level studies indicate that cigarette smoking promotes immune phenotypes resembling accelerated aging, including contraction of naïve lymphocyte pools, expansion of senescent T-cell populations, disruption of regulatory signaling pathways, and persistent remodeling of adaptive immunity.

At the cellular level, smoking has been associated with reductions in naïve T-cell populations and expansion of terminally differentiated or senescent T-cell subsets. Experimental and high-dimensional immune profiling studies provide further support for the ability of smoking exposure to induce hallmarks of immunosenescence. Single-cell RNA sequencing and mass cytometry analyses of peripheral blood from smokers have demonstrated expansion of differentiated CD16^+^ CD8^+^ T-cell populations, reduced naïve T-cell representation, and transcriptional programs consistent with altered cytotoxic function and immune aging (64). Complementary observational and immunophenotyping studies further show that smokers exhibit T-cell distributions resembling those of older individuals, including shifts toward more differentiated and senescent phenotypes (65). Age-associated exhausted T-cell populations characterized by increased expression of inhibitory receptors such as PD-1 and Tim-3 further support the link between chronic immune activation and senescent immune remodeling (68). More recent systems-level analyses indicate that smoking produces persistent alterations in adaptive immune responses, consistent with durable immune reprogramming after exposure (66). Together, these findings support the interpretation that smoking can promote immunosenescence-like immune remodeling, although direct longitudinal evidence in MS-specific cohorts remains limited. Importantly, antigen-driven senescent CD4^+^ T-cell populations have also been characterized directly in people with MS, supporting the presence of adaptive immune exhaustion and senescence within disease-relevant immune responses (67).

Smoking also influences immune regulation at a molecular signaling level. Components of the cholinergic system — including choline transporters and acetylcholine receptors — are expressed on immune cells and contribute to regulation of inflammatory responses (72, 73). Experimental work has shown that choline transporter activity in T lymphocytes affects cytokine production and immune activation states (62), and complementary studies demonstrate that choline-transporter–related pathways more broadly regulate T-cell effector function (63).

Systems-level immunologic analyses further demonstrate that smoking reshapes immune responses across both innate and adaptive pathways. In a large cohort study of healthy individuals, smoking emerged as a major determinant of cytokine response variability, with effects comparable in magnitude to age and genetic factors. Notably, while smoking-associated alterations in innate immune responses largely resolved after cessation, adaptive immune changes persisted long after individuals quit smoking, consistent with epigenetic reprogramming of immune cells (66). Similarly, large immunologic datasets in otherwise healthy adults identify smoking as a major determinant of immune variability alongside age and socioeconomic exposures, reinforcing the concept that smoking shapes immune function at a systemic level (74). Dysregulation of these pathways through chronic exposure to cigarette smoke or nicotine may therefore impair immune homeostasis and promote persistent inflammatory signaling, further contributing to immunosenescence-like patterns in people with MS.

Beyond cellular composition and signaling, smoking is linked with systemic inflammatory activation and oxidative stress, both recognized accelerants of immune aging. Persistent exposure to smoke-related inflammatory stimuli can drive repeated immune activation and antigenic load, conditions known to hasten the transition from functional memory responses toward senescent immune phenotypes (75). This cumulative burden may intersect with MS-related immune dysregulation, amplifying immune-aging processes already present in the disease.

**Figure 1:**
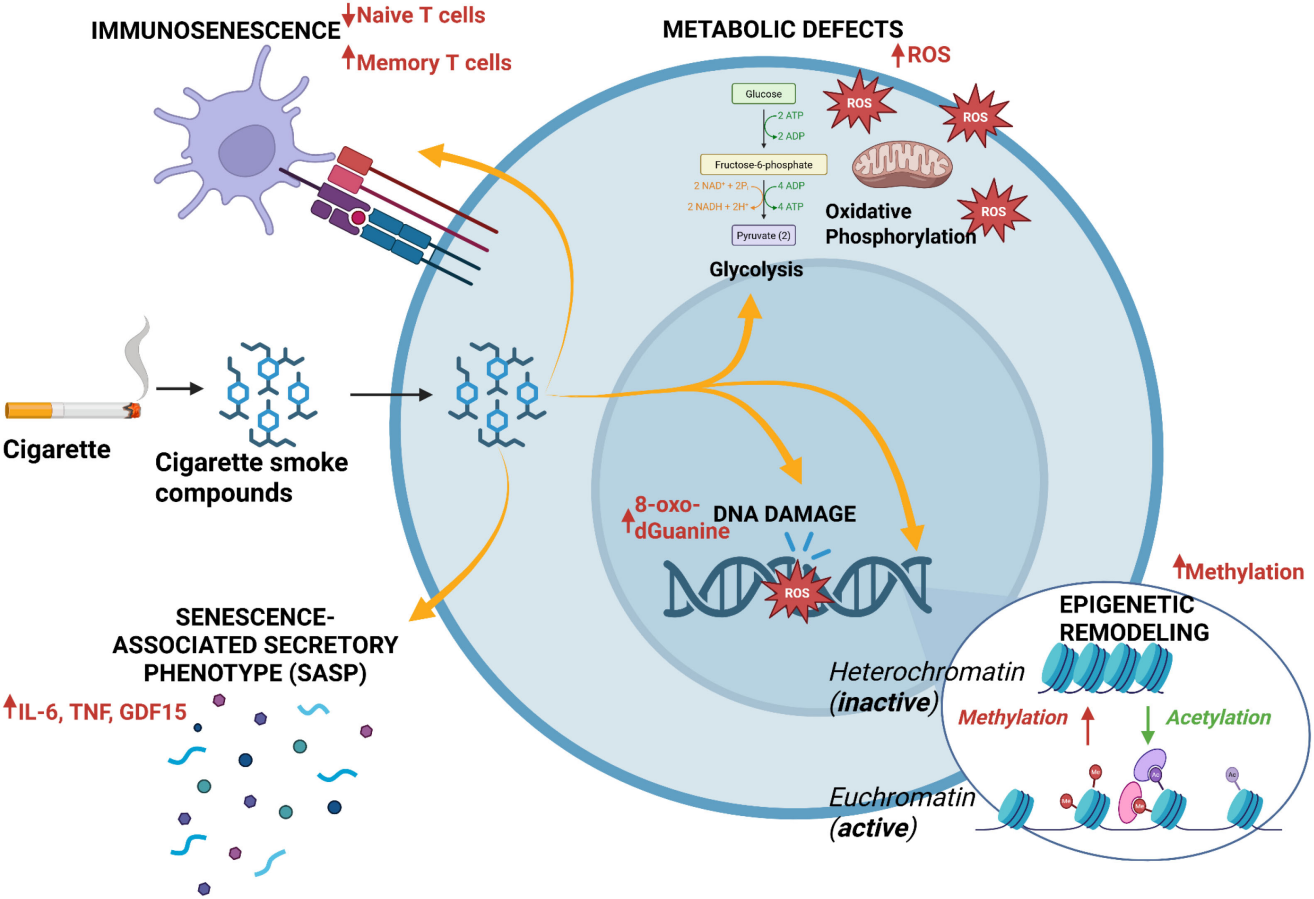
How smoking may influence molecular and cellular events to modify immune mechanisms in MS. This image illustrates molecular- and cell-based mechanisms leading to defective immune activity in MS patients. Smoking contains >7000 chemicals that directly create DNA/epigenetic modifications and manifests at the cellular level - as increased ROS production, SASP output, and inflammatory cytokine production. Ultimately, these may contribute to the immune defects observed in MS. Created in BioRender. Mytych, J. (2026) https://BioRender.com/clppiyw.

Together, these observations suggest that smoking may not simply act as a pro-inflammatory trigger, but rather as a driver of premature immune aging. The proposed biological pathway linking smoking exposure, immunosenescence features, and MS pathophysiology is summarized in **Table 2**. By inducing senescent-like immune profiles, reducing naïve lymphocyte pools, and altering regulatory signaling pathways, smoking creates an immune environment characterized by diminished adaptability and persistent, low-grade inflammation (62, 65). Within the context of MS, such changes could promote both heightened early inflammatory activity and impaired capacity for long-term immune regulation and repair. These biological insights provide a mechanistic foundation for clinical observations linking smoking to worse MS outcomes and set the stage for understanding how smoking-driven immune aging may shape disease course. These mechanistic interactions are summarized in **Table 2** and **Figure 1**, which outlines how smoking-driven immune changes parallel key features of immunosenescence and may influence MS biology.

### 3.1 Tobacco smoke components driving immunosenescence and MS pathobiology

Cigarette smoke is a complex mixture of thousands of chemicals, including reactive oxidants, aldehydes, polycyclic aromatic hydrocarbons (PAHs), particulate matter, heavy metals, and nicotine (76). While many studies consistently link smoking to altered immune function and worse outcomes in MS, the specific components responsible for these effects are not completely defined. Emerging evidence suggests that non-nicotine combustion byproducts are the primary drivers of immune dysregulation, acting through pathways that converge on cellular stress, epigenetic remodeling, and chronic immune activation, which are hallmarks of immunosenescence.

A major contributor to these processes is oxidative and DNA-damage inducing stress. Reactive oxygen species and aldehydes (especially acrolein) induce DNA damage, mitochondrial dysfunction, and telomere shortening, thereby promoting cellular senescence pathways (21, 22, 77). In immune cells, these stressors impair proliferative capacity and allow the accumulation of terminally differentiated/senescent T-cell subsets (78, 79). Mitochondrial damage may further amplify these effects through release of damage-associated molecular patterns (DAMPs) (80), which will reinforce chronic inflammatory signaling and contribute to the low-grade inflammatory state characteristic of immune aging.

Additional smoke constituents, including PAHs, particulate molecules, and heavy metals, can further drive immune aging. PAHs act engage aryl hydrocarbon receptor (AHR) signaling to influence T-cell differentiation and epigenetic programs (81). Particulate matter and metals promote persistent activation of innate immune cells (82, 83), including macrophage and microglial activation. Together, the exposure to these smoke constituents sustain chronic, dysregulated immune activation and may contribute to compartmentalized CNS inflammation, impaired repair, and progressive neurodegeneration in MS.

In contrast, nicotine alone is unlikely to be a primary driver of immunosenescence or MS risk. Nicotine primarily exerts immunomodulatory effects through cholinergic signaling pathways that can dampen inflammatory responses and reduce immune activation (84). Importantly, epidemiologic studies suggest that not all nicotine-containing exposures confer equivalent MS risk (**Table 3**). For example, oral tobacco products (e.g., snuff) have not been associated with the same degree of MS risk or progression as cigarette smoking (85). However, this distinction should be interpreted cautiously, as oral tobacco differs substantially from inhaled non-combustible products such as e-cigarettes and heated tobacco devices in terms of exposure route, pharmacokinetics, and toxicant profiles. The effects of these inhaled non-combustible products on immune aging and MS biology remain incompletely defined. These observations support the idea that combustion-derived toxicants, rather than nicotine itself, are the principal mediators of smoking-associated immune aging and disease progression.

**Table 3:** Epidemiologic studies of cigarette smoking and multiple sclerosis risk and disease course.

Disease	Study design	No. cases/controls (or sample size)	Smoking exposure evaluated	Study duration/region	Impact of smoking on disease or immune aging	Key reference
Disease activity and progression	Population-based longitudinal cohort	9,089 MS patients	Current smoking, passive smoking, smoking cessation, and oral tobacco (snuff) use	Sweden; up to 15 years follow-up	Current and passive smoking were associated with faster disability progression and worse clinical outcomes in MS, while smoking cessation was associated with more favorable outcomes. Snuff use was associated with slower EDSS progression compared with never-users.	(85)
Disability progression after smoking cessation	Retrospective and prospective cohort study	7,983 MS patients	Current smoking, former smoking, and smoking cessation	United Kingdom MS Register, 2011–2020	Current smoking was associated with faster disability worsening and poorer patient-reported outcomes, while smoking cessation was associated with slowing of motor disability progression to rates similar to never-smokers.	(86)
MS conversion (CIS → clinically definite MS)	Prospective cohort	250 CIS patients	Smoking status at time of CIS	Netherlands; median follow-up 58 months	Smoking at the time of clinically isolated syndrome was independently associated with increased risk of conversion to clinically definite MS, suggesting that tobacco exposure may accelerate early disease development.	(87)
Disability progression	Longitudinal cohort	895 MS patients	Current vs former vs never smoking	United Kingdom; median follow-up 8 years	Smoking was associated with more rapid disability accumulation and faster progression along the EDSS, indicating a negative effect on long-term neurologic outcomes.	(4)
MS susceptibility	Prospective cohort	902 cases; 1,855 controls	Ever vs never smoking	Sweden, 1991–2008	Smoking was associated with a significantly increased risk of developing MS, with evidence of a dose–response relationship indicating that cumulative tobacco exposure contributes to disease susceptibility.	(2)
MS conversion (CIS → MS)	Case–control	129 participants	Current and former smoking	Austria, 2003–2007	Smokers demonstrated faster conversion from clinically isolated syndrome (CIS) to clinically definite MS compared with nonsmokers, suggesting that smoking accelerates early disease development.	(3)
Disease progression (SPMS transition)	Prospective cohort	52 MS cases; 30 controls	Pack-years and current smoking	United States	Greater cumulative smoking exposure was associated with earlier transition to secondary progressive MS (SPMS), supporting a role for smoking in accelerating disease progression.	(1)

This table summarizes key epidemiologic and longitudinal cohort studies examining the relationship between cigarette smoking and multiple sclerosis (MS), including disease susceptibility, conversion from clinically isolated syndrome (CIS) to clinically definite MS, disability accumulation, and progression to secondary progressive disease. Collectively, these findings support smoking as a modifiable factor associated with worse clinical outcomes and accelerated disease evolution in MS.

In addition to traditional combustible cigarettes, emerging non-combustible tobacco products such as e-cigarettes and heated tobacco devices have gained widespread use. Although these products eliminate combustion, growing evidence suggests that they are not biologically inert. Experimental and clinical studies indicate that e-cigarette aerosols can alter innate and adaptive immune responses, promote oxidative stress, and influence inflammatory signaling pathways (88). Some data also suggest potential effects on neuroinflammatory processes, although these findings remain limited and are not yet well characterized in the context of multiple sclerosis. Importantly, the long-term impact of these products on immune aging and disease progression remains incompletely understood. However, much of the available evidence remains associative, and direct longitudinal studies linking smoking exposure to immunosenescence trajectories in MS are limited. As use of these devices continues to increase, further research will be essential to clarify their role in immune dysregulation and MS biology.

## 4 Integrating smoking-driven immunosenescence with MS course and progression

The relationships among cigarette smoking, immunosenescence, and multiple sclerosis (MS) appear to converge on a common biological theme: a progressive shift from flexible, well-regulated immune responses toward chronically activated, less adaptable immune states. Taken together, the available evidence suggests that smoking influences MS through both early inflammatory and later immune-aging pathways: it may enhance susceptibility and relapse activity through pro-inflammatory, oxidative, and epigenetic mechanisms, while simultaneously accelerating immunosenescence-related processes that favor progression and chronic neurodegeneration. This shift is consistent with both age-related immune remodeling and with the immune alterations observed in people who smoke. When layered on top of the immune dysregulation intrinsic to MS, smoking-induced immune aging may amplify disease susceptibility, accelerate progression, and diminish the capacity for tissue repair. **Figure 1** illustrates this conceptual pathway, outlining how smoking-related immune aging may interact with MS disease mechanisms across the lifespan. Although the immunosenescence framework aligns most directly with progression, the association of smoking with MS susceptibility and relapse activity across diverse populations indicates that its biologic effects extend beyond inflammaging alone (89).

Epidemiologic studies demonstrate that smoking increases the risk of developing MS and is associated with a more inflammatory and aggressive disease course, including higher relapse activity, earlier disability accumulation, greater long-term brain atrophy and cognitive decline, and faster conversion to secondary progressive MS (90). Senescent lymphocytes exhibit diminished proliferative capacity and skewed cytokine production, fostering a milieu of chronic, low-grade inflammation. Within the CNS, such persistent inflammatory signaling may contribute to microglial activation, impaired debris clearance, and progressive neuroaxonal injury, processes that are already central to MS pathology (60, 91).

Smoking-associated alterations in immune regulation may further influence the balance between relapse-driven inflammation and progressive, compartmentalized CNS damage. Immunosenescence has been proposed as one explanation for the paradox in MS whereby relapse activity declines with age even as disability continues to worsen. As immune diversity contracts and regulatory networks weaken, inflammatory responses may become less acutely aggressive yet chronically sustained, favoring neurodegenerative mechanisms over overt relapses (16, 58, 91, 92), with recent work further supporting the presence of progression-related immune remodeling consistent with accelerated immune aging in MS (25). Because smoking appears to accelerate similar aging-like immune trajectories, individuals with MS who smoke may reach this transition point earlier than nonsmokers. In the setting of MS, where inflammatory circuits are already sensitized, such alterations could further impair regulation and promote smoldering CNS inflammation.

Smoking-related immune aging may also have implications for therapeutic responsiveness. Many disease-modifying therapies (DMTs) primarily target adaptive immune activation. If smoking accelerates contraction of naïve lymphocyte pools and expansion of exhausted or senescent populations, the biological substrate on which these therapies act may be altered. Although data are still emerging, observational studies suggest that smokers may derive less benefit from some DMTs and may experience more rapid disability accumulation despite treatment, while smoking cessation may partially attenuate disability worsening over time, consistent with a shift toward mechanisms less dependent on peripheral immune activation (4, 86, 93, 94). These patterns underscore the importance of considering smoking status when interpreting treatment outcomes and planning long-term management, particularly given more recent evidence that smoking may influence disease activity even among patients receiving oral disease-modifying therapies such as fingolimod and dimethyl fumarate (95).

**Figure 2:**
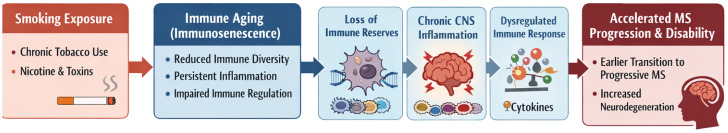
How smoking accelerates immune aging and MS. This image illustrates the proposed pathway through which cigarette smoking may promote immunosenescence, leading to reduced immune diversity, persistent low-grade inflammation, and impaired immune regulation. These immune-aging processes may, in turn, contribute to increased MS susceptibility, more aggressive early disease activity, and earlier transition to progressive neurodegeneration.

Taken together, current evidence supports a conceptual model in which smoking amplifies immunosenescence-related processes relevant to MS. By promoting premature immune aging, smoking may increase initial susceptibility to disease, accelerate the transition from inflammatory relapsing activity to progressive neurodegeneration, and potentially attenuate therapeutic efficacy. Although causality cannot yet be fully established, the convergence of epidemiologic, immunologic, and mechanistic findings provides a compelling rationale for viewing smoking not only as a risk factor, but as a biologic modifier of MS trajectory. Continued research that integrates biomarkers of immune aging, longitudinal clinical outcomes, and intervention studies — including smoking cessation — will be essential to clarify these relationships and identify modifiable targets along the disease course. Additionally, heterogeneity in study design, smoking exposure definitions, and immune phenotyping approaches complicates direct comparison across studies. As illustrated in **Figure 2**, smoking may shift immune-aging trajectories leftward, meaning that biological aging processes relevant to MS occur earlier in individuals who smoked.

## 5 Clinical and therapeutic implications

**Figure 3:**
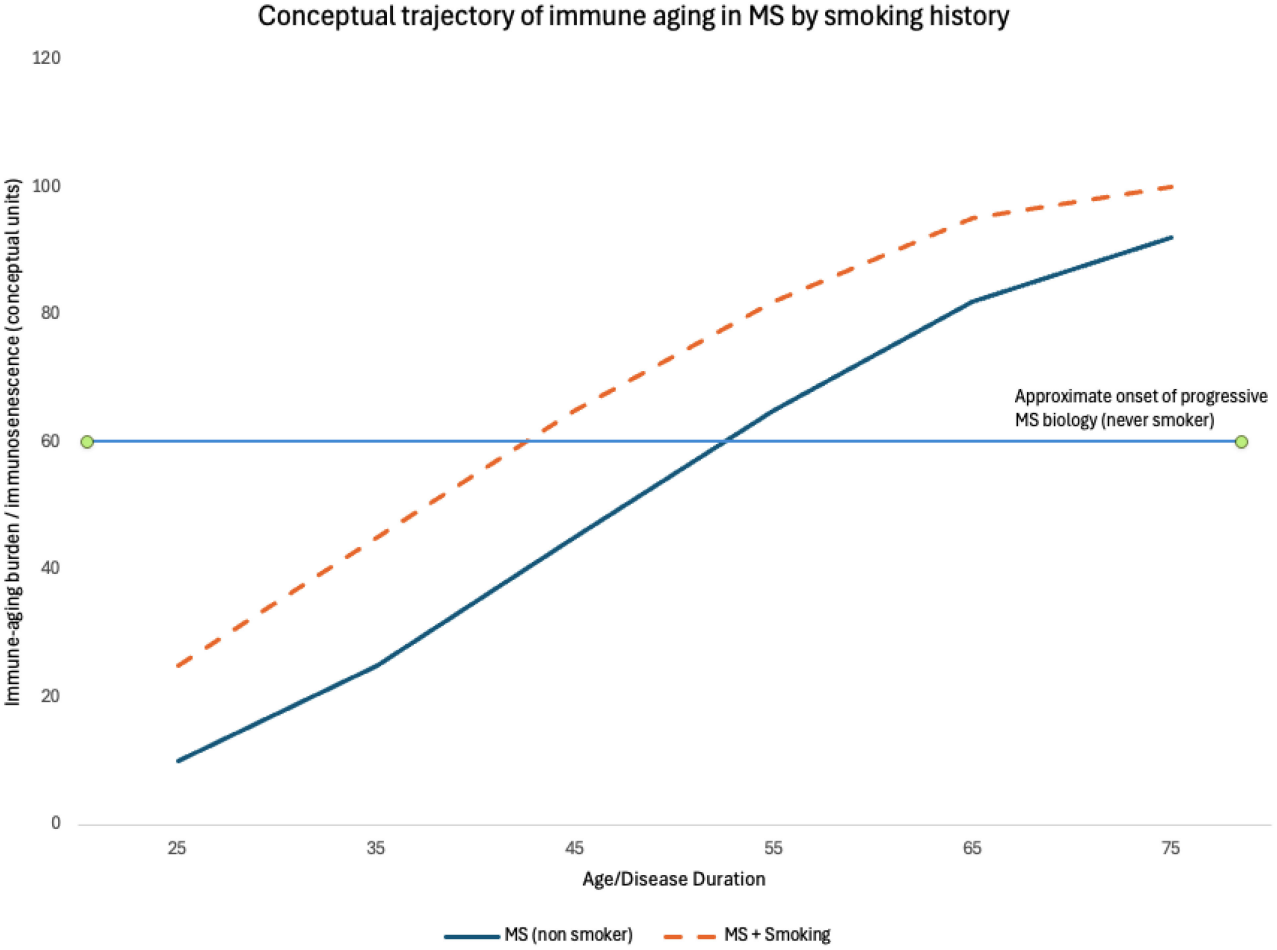
Conceptual trajectory of immune aging in MS. The diagram depicts a conceptual comparison between normal immune aging and smoking-accelerated immune aging. In individuals who smoke, immune function may decline more rapidly, potentially shifting the onset of progressive MS biology earlier in the disease course. The threshold shown is conceptual and intended to illustrate trajectory differences rather than exact clinical timing.

The intersection of smoking, immunosenescence, and MS has important implications for clinical care, patient counseling, and therapeutic decision-making. Recognizing smoking as a biologic modifier of immune aging reframes it not only as a lifestyle risk factor, but also as a potentially actionable contributor to disease progression and treatment response (**Figure 3**).

From a risk-reduction perspective, smoking cessation remains one of the most powerful modifiable behaviors available to people with MS. Epidemiologic evidence links active smoking to increased MS risk, accelerated disability accumulation, and earlier transition to secondary progressive disease (4, 93, 96). Framing smoking in the context of premature immune aging may enhance counseling messages, helping patients understand why even relatively brief smoking exposure can have long-term immunologic consequences. Given the strong parallels between smoking-related immune remodeling and age-associated immunosenescence (64, 65), cessation efforts may help prevent or slow the emergence of senescent immune phenotypes that contribute to progressive disease biology. **Table 4** summarizes key clinical outcomes associated with smoking in MS and highlights how these findings inform patient counseling and treatment planning.

**Table 4:** Clinical outcomes associated with smoking in multiple sclerosis.

Outcome domain	Summary of evidence	Clinical interpretation	Key references
MS susceptibility	Current and past smokers have higher risk of developing MS, with dose–response effects and risk reduction after cessation.	Smoking likely contributes to MS onset through immune and molecular pathways.	(2, 89)
Early disease activity and conversion	Smoking is associated with increased inflammatory activity and faster conversion from CIS to clinically definite MS early in disease.	Smoking may amplify early adaptive immune activation and accelerate disease evolution.	(2, 87)
Disability progression	Smokers show faster disability accumulation compared with nonsmokers in longitudinal cohorts.	Smoking may promote neurodegeneration and chronic inflammation.	(85, 86, 90)
Conversion to Secondary Progressive MS (SPMS)	Smoking is linked to earlier transition to SPMS.	Consistent with accelerated immune aging and loss of repair capacity.	(3, 93, 97)
Response to disease-modifying therapy (DMT)	Smokers may derive less benefit from some DMTs and progress despite treatment.	Immune aging and smoldering pathology may limit treatment responsiveness.	(94, 95)
Smoking cessation	Smoking cessation after MS diagnosis is associated with slower disability worsening, with motor progression rates approaching those of never-smokers over time.	Smoking is a modifiable risk factor, and cessation may alter long-term disability trajectory.	(86)

Evidence from observational studies indicates that cigarette smoking is associated with increased MS risk, greater early-disease activity, faster disability accumulation, and earlier transition to secondary progressive MS.

Smoking status may also be clinically relevant when selecting and monitoring disease-modifying therapies (DMTs). Observational data suggest that smokers may have poorer clinical outcomes despite treatment, including higher disability progression rates (4, 93, 94). Although causality has not been conclusively established, incorporating smoking status into therapeutic discussions and shared decision-making may help set realistic expectations regarding treatment response and emphasize the additional benefits of cessation.

Mechanistically, smoking may also interfere with endogenous immune-regulatory pathways. The presence of a functional cholinergic system within immune cells (98) and the role of choline transporters in shaping T-cell activation thresholds (62) suggest that chronic nicotine exposure could alter inflammatory tone and immune resilience. These insights point to the possibility that smoking cessation may not only remove an inflammatory trigger but also help restore regulatory signaling pathways that are critical for maintaining immune balance in MS.

An additional implication involves aging itself. Because MS progression increasingly reflects neurodegenerative and compartmentalized inflammatory processes with advancing age (16, 60, 99, 100), individuals who smoke may reach age-related transition points earlier as a result of smoking-accelerated immunosenescence. Clinicians may therefore need to adjust long-term disease monitoring strategies for smokers, with heightened vigilance for early markers of progression and more proactive timing of therapeutic escalation.

Finally, these relationships have relevance for multidisciplinary care. Integration of smoking cessation programs into MS clinics, collaboration with behavioral health services, and reinforcement of cessation counseling by neurologists, nurses, and rehabilitation professionals may leverage the full spectrum of patient contact to reduce smoking-related disease burden. Importantly, patients should be reassured that cessation remains beneficial even after disease onset and at all stages of MS.

**Figure 4:**
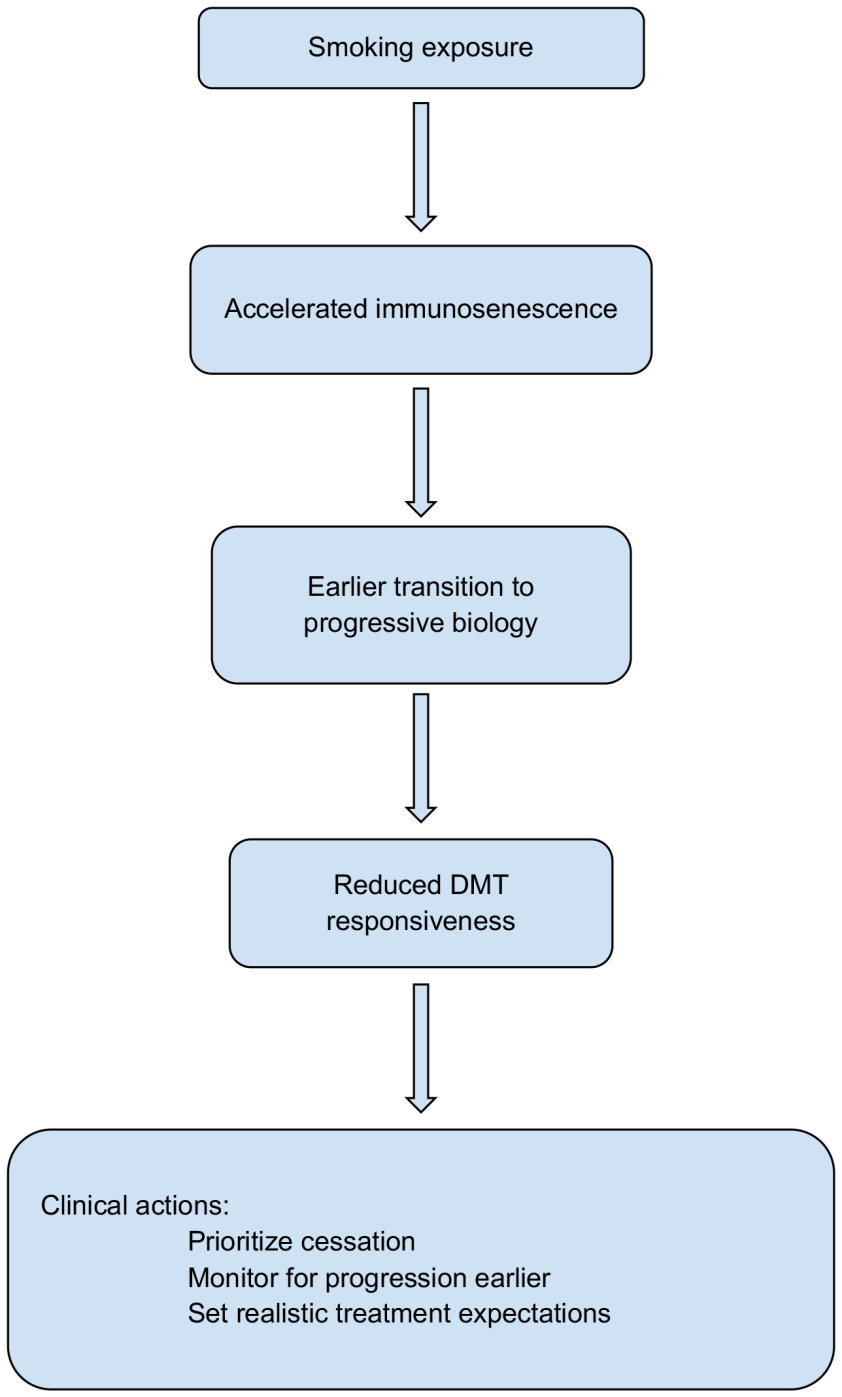
Clinical implications of smoking-related immune aging in MS. This image summarizes potential clinical consequences of smoking-associated immunosenescence, including earlier progression, reduced responsiveness to disease-modifying therapies, and increased overall disease burden. It highlights key clinical actions such as prioritizing smoking cessation counseling, monitoring earlier for signs of progression, and setting realistic expectations regarding treatment outcomes.

In summary, viewing smoking through the lens of immunosenescence underscores its role as both a risk factor and a biologic modifier of MS. This perspective strengthens the rationale for aggressive smoking cessation counseling, supports consideration of smoking status in therapeutic planning and highlights the need to better understand how immune aging influences treatment response and disease evolution across the lifespan (**Figure 4**).

## 6 Future directions and research priorities

Although growing evidence links smoking, immunosenescence, and MS, many aspects of this relationship remain incompletely defined. Future work will benefit from moving beyond association studies toward mechanistic and longitudinal research that can clarify causality, identify biomarkers of immune aging, and evaluate the effects of targeted interventions.

One priority is the development and validation of standardized biomarkers of immunosenescence that are feasible for use in MS cohorts. Current measures — such as shifts in naïve-to-memory T-cell ratios, accumulation of senescent or “exhausted” T-cell subsets, epigenetic aging signatures, and markers of chronic microglial activation — have been studied individually, but few have been systematically applied to MS populations across disease stages. Integrating these markers into longitudinal studies would allow investigators to determine whether smoking accelerates immune aging trajectories in patients, and whether such changes predict clinical outcomes including relapse frequency, disability progression, and neurodegeneration.

A second area of need involves disentangling the temporal sequence of smoking exposure, immune remodeling, and disease evolution. Most existing studies assess smoking status at a single time point. Prospective studies that repeatedly measure smoking behavior, pulmonary exposures, and immune-aging markers — including after cessation — could clarify whether immunosenescence mediates the harmful effects of smoking and whether these processes can be slowed or reversed. These studies will also need to account for interactions among smoking, biological aging, sex, genetics, and comorbidities that influence immune trajectories.

Interventional research is equally important. If smoking accelerates immune aging in MS, then smoking cessation may represent not only a lifestyle recommendation, but a biologically targeted therapy. Trials or pragmatic observational studies that embed structured cessation programs within MS care, while concurrently tracking immunologic and radiologic outcomes, could determine whether cessation modifies immune aging pathways or alters disease progression. Such approaches may also clarify the time window during which cessation confers the greatest benefit.

Finally, the concept of immunosenescence raises broader questions about treatment optimization across the MS lifespan. As the disease increasingly reflects neurodegenerative and compartmentalized inflammatory processes with advancing age, it will be essential to understand how smoking-related immune aging intersects with therapeutic mechanisms of action. Future work should explore whether specific disease-modifying therapies differentially benefit individuals with accelerated immune aging, and whether markers of immunosenescence can guide personalized therapy selection.

In sum, advancing the field will require a coordinated effort that integrates immunology, epidemiology, neuroimaging, and clinical trial methodology. By clarifying how smoking shapes immune aging and MS biology, future research has the potential to identify novel targets for intervention and reinforce smoking cessation as a central component of comprehensive MS care.

## 7 Conclusion

The convergence of evidence across epidemiologic, immunologic, and mechanistic studies suggests that cigarette smoking influences MS not only as an environmental risk factor, but also as a biologic modifier of disease biology. By promoting immune remodeling that mirrors and potentially accelerates immunosenescence, smoking may shape susceptibility to MS, hasten the transition from relapsing inflammatory activity to progressive neurodegeneration, and reduce the effectiveness of existing therapies. These relationships highlight immune aging as a useful framework for interpreting the diverse effects of smoking on MS.

Understanding smoking within this context shifts the clinical conversation. Rather than focusing solely on relapse prevention or short-term outcomes, counseling can emphasize the long-term consequences of smoking on immune resilience, brain health, and aging trajectories. At the same time, continued research is needed to define biomarkers of immune aging, determine whether smoking cessation can modify these pathways, and clarify how immunosenescence should inform therapeutic planning across the lifespan.

Altogether, viewing smoking through the lens of immune aging strengthens the rationale for aggressive cessation efforts, supports more personalized treatment strategies, and opens avenues for investigating novel therapeutic targets. Taken together, these data suggest that smoking acts as a broad biologic modifier of MS, influencing disease susceptibility, inflammatory activity, and long-term progression through overlapping but potentially distinct mechanisms. Integrating smoking, immunosenescence, and MS within a single conceptual model may ultimately help clinicians and researchers better understand disease heterogeneity — and improve long-term outcomes for people living with MS.

## References

[1] Hernán M Jick SS Logroscino G Olek MJ Ascherio A Jick H . Cigarette smoking and the progression of multiple sclerosis. Brain: A J Neurol. (2005) 128:1461–7. doi: 10.1093/brain/awh471 15758034

[2] Hedstrom AK Bäärnhielm M Olsson T Alfredsson L . Tobacco smoking, but not Swedish snuff use, increases the risk of multiple sclerosis. Neurology. (2009) 73:696–701. doi: 10.1212/WNL.0b013e3181b59c40 19720976

[3] DiPauli F Reindl M Ehling R Schautzer F Gneiss C Lutterotti A . Smoking is a risk factor for early conversion to clinically definite multiple sclerosis. Multiple Sclerosis (Houndmills Basingstoke England). (2008) 14:1026–30. doi: 10.1177/1352458508093679 18632775

[4] Manouchehrinia A Tench CR Maxted J Bibani RH Britton J Constantinescu CS . Tobacco smoking and disability progression in multiple sclerosis: United Kingdom cohort study. Brain A J Neurol. (2013) 136:2298–304. doi: 10.1093/brain/awt139 23757766 PMC3692034

[5] Marabita F Almgren M Sjöholm LK Kular L Liu Y James T . Smoking induces DNA methylation changes in multiple sclerosis patients with exposure-response relationship. Sci Rep. (2017) 7:14586. doi: 10.1038/s41598-017-14788-w 29109506 PMC5674007

[6] Faist B Fleischer B Jacobsen M . Cytomegalovirus infection - and age-dependent changes in human CD8+ T-cell cytokine expression patterns. Clin Vaccine Immunol CVI. (2010) 17:986–92. doi: 10.1128/CVI.00455-09 20427631 PMC2884431

[7] Yin F Li J Gao Y . Immune cell senescence in autoimmunity: implications for disease pathogenesis and therapeutic targeting. Front Immunol. (2025) 16:1596686. doi: 10.3389/fimmu.2025.1596686 40852730 PMC12367673

[8] Fagnoni FF Vescovini R Passeri G Bologna G Pedrazzoni M Lavagetto G . Shortage of circulating naive CD8(+) T cells provides new insights on immunodeficiency in aging. Blood. (2000) 95:2860–8. doi: 10.1182/blood.v95.9.2860.009k35_2860_2868 10779432

[9] Weng NP Levine BL June CH Hodes RJ . Human naive and memory T lymphocytes differ in telomeric length and replicative potential. PNAS. (1995) 92:11091–4. doi: 10.1073/pnas.92.24.11091 7479943 PMC40577

[10] Goodwin K Viboud C Simonsen L . Antibody response to influenza vaccination in the elderly: a quantitative review. Vaccine. (2006) 24:1159–69. doi: 10.1016/j.vaccine.2005.08.105 16213065

[11] Thewissen M Somers V Hellings N Fraussen J Damoiseaux J Stinissen P . Premature immunosenescence in multiple sclerosis and its association with chronic cytomegalovirus infection. J Immunol. (2005) 174(11):7160–67. doi: 10.4049/jimmunol.174.11.7160 15905560

[12] Duszczyszyn DA Williams JL Mason H Lapierre Y Antel J Haegert DG . Thymic involution and proliferative T-cell responses in multiple sclerosis. J Neuroimmunol. (2010) 221:73–80. doi: 10.1016/j.jneuroim.2010.02.005 20223525

[13] Haegert DG Hackenbroch JD Duszczyszyn D Fitz-Gerald L Zastepa E Mason H . Reduced thymic output and peripheral naïve CD4 T-cell alterations in primary progressive multiple sclerosis (PPMS). J Neuroimmunol. (2011) 233:233–9. doi: 10.1016/j.jneuroim.2010.12.007 21272945

[14] Haegele KF Stueckle CA Malin JP Sindern E . Increase of CD8+ T-effector memory cells in peripheral blood of patients with relapsing-remitting multiple sclerosis compared to healthy controls. J Neuroimmunol. (2007) 183:168–74. doi: 10.1016/j.jneuroim.2006.09.008 17084910

[15] Balint B Haas J Schwarz A Jarius S Fürwentsches A Engelhardt K . -cell homeostasis in pediatric multiple sclerosis: old cells in young patients. Neurology. (2013) 81:784–92. doi: 10.1212/WNL.0b013e3182a2ce0e 23911752

[16] Scalfari A Neuhaus A Degenhardt A Rice GP Muraro PA Daumer M . Onset of secondary progressive phase and long-term evolution of multiple sclerosis. J Neurol Neurosurg Psychiat. (2014) 85(1):67–75. doi: 10.1136/jnnp-2012-304333 23486991

[17] University of California, San Francisco MS-EPIC Team Cree BAC Gourraud PA Oksenberg JR Bevan C Crabtree-Hartman E . Long-term evolution of multiple sclerosis disability in the treatment era. Annals Neurol. (2016) 80(4):499–510. doi: 10.1002/ana.24747 PMC5105678 27464262

[18] Müller L Benedetto S . Immunosenescence and inflammaging: Mechanisms and modulation through diet and lifestyle. Front Immunol. (2025) 16:1708280. doi: 10.3389/fimmu.2025.1708280 41425546 PMC12711513

[19] Qiu F Liang CL Liu H Zeng YQ Hou S Huang S . Impacts of cigarette smoking on immune responsiveness: Up and down or upside down? Oncotarget. (2017) 8:268–84. doi: 10.18632/oncotarget.13613 27902485 PMC5352117

[20] Piaggeschi G Rolla S Rossi N Brusa D Naccarati A Couvreur S . Immune trait shifts in association with tobacco smoking: A study in healthy women. Front Immunol. (2021) 12:637974. doi: 10.3389/fimmu.2021.637974 33767708 PMC7985448

[21] Valavanidis A Vlachogianni T Fiotakis K . Tobacco smoke: involvement of reactive oxygen species and stable free radicals in mechanisms of oxidative damage, carcinogenesis and synergistic effects with other respirable particles. Int J Environ Res Public Health. (2009) 6:445–62. doi: 10.3390/ijerph6020445 19440393 PMC2672368

[22] Weng MW Lee HW Park SH Hu Y Wang HT Chen LC . Aldehydes are the predominant forces inducing DNA damage and inhibiting DNA repair in tobacco smoke carcinogenesis. PNAS. (2018) 115:E6152–61. doi: 10.1073/pnas.1804869115 29915082 PMC6142211

[23] Lindahl T . Instability and decay of the primary structure of DNA. Nature. (1993) 362:709–15. doi: 10.1038/362709a0 8469282

[24] Sun L Wu J Du F Chen X Chen ZJ . Cyclic GMP-AMP synthase is a cytosolic DNA sensor that activates the type I interferon pathway. Sci (New York NY). (2013) 339:786–91. doi: 10.1126/science.1232458 23258413 PMC3863629

[25] Maltby V Xavier A Ewing E Campagna MP Sampangi S Scott RJ . Evaluation of cell-specific epigenetic age acceleration in people with multiple sclerosis. Neurology. (2023) 101:e679–89. doi: 10.1212/wnl.0000000000207489 37541839 PMC10437016

[26] International Multiple Sclerosis Genetics Consortium . Multiple sclerosis genomic map implicates peripheral immune cells and microglia in susceptibility. Sci (New York NY). (2019) 365:eaav7188. doi: 10.1126/science.aav7188 31604244 PMC7241648

[27] Ramirez JM Ribeiro R Soldatkina O Moraes A García-Pérez R Oliveros W . The molecular impact of cigarette smoking resembles aging across tissues. Genome Med. (2025) 17:66. doi: 10.1186/s13073-025-01485-x 40457411 PMC12131351

[28] Ferrucci L Corsi A Lauretani F Bandinelli S Bartali B Taub DD . The origins of age-related proinflammatory state. Blood. (2005) 105:2294–9. doi: 10.1182/blood-2004-07-2599 15572589 PMC9828256

[29] Kritchevsky SB Cesari M Pahor M . Inflammatory markers and cardiovascular health in older adults. Cardiovasc Res. (2005) 66:265–75. doi: 10.1016/j.cardiores.2004.12.026 15820195

[30] Kalair A Pavan M Alpert N Ghaffari S Taioli E . Blood inflammatory markers and mortality in the US population: A Health and Retirement Survey (HRS) analysis. PLoS One. (2023) 18:e0293027. doi: 10.1371/journal.pone.0293027 37844090 PMC10578595

[31] Frasca D Landin AM Lechner SC Ryan JG Schwartz R Riley RL . Aging down-regulates the transcription factor E2A, activation-induced cytidine deaminase, and Ig class switch in human B cells. J Immunol (Baltimore Md 1950). (2008) 180:5283–90. doi: 10.4049/jimmunol.180.8.5283 18390709

[32] de Mol JD Kuiper J Tsiantoulas D Foks AC . The Dynamics of B Cell Aging in Health and Disease. Front Immunol. (2021) 12:733566. doi: 10.3389/fimmu.2021.733566 34675924 PMC8524000

[33] Streit WJ Sammons NW Kuhns AJ Sparks DL . Dystrophic microglia in the aging human brain. Glia. (2004) 45:208–12. doi: 10.1002/glia.10319 14730714

[34] Hickman SE Kingery ND Ohsumi TK Borowsky ML Wang LC Means TK . The microglial sensome revealed by direct RNA sequencing. Nat Neurosci. (2013) 16:1896–905. doi: 10.1038/nn.3554 24162652 PMC3840123

[35] Dilger RN Johnson RW . Aging, microglial cell priming, and the discordant central inflammatory response to signals from the peripheral immune system. J Leukocyte Biol. (2008) 84:932–9. doi: 10.1189/jlb.0208108 18495785 PMC2538600

[36] Galatro TF Holtman IR Lerario AM Vainchtein ID Brouwer N Sola PR . Transcriptomic analysis of purified human cortical microglia reveals age-associated changes. Nat Neurosci. (2017) 20:1162–71. doi: 10.1038/nn.4597 28671693

[37] Patel T Carnwath TP Wang X Allen M Lincoln SJ Lewis-Tuffin LJ . Transcriptional landscape of human microglia implicates age, sex, and APOE-related immunometabolic pathway perturbations. Aging Cell. (2022) 21:e13606. doi: 10.1111/acel.13606 35388616 PMC9124307

[38] Basisty N Kale A Jeon OH Kuehnemann C Payne T Rao C . A proteomic atlas of senescence-associated secretomes for aging biomarker development. PLoS Biol. (2020) 18:e3000599. doi: 10.1371/journal.pbio.3000599 31945054 PMC6964821

[39] Hernandez-Segura A de Jong TV Melov S Guryev V Campisi J Demaria M . Unmasking transcriptional heterogeneity in senescent cells. Curr Biol: CB. (2017) 27:2652–2660.e4. doi: 10.1016/j.cub.2017.07.033 28844647 PMC5788810

[40] Tylutka A Walas Ł Zembron-Lacny A . Level of IL-6, TNF, and IL-1β and age-related diseases: a systematic review and meta-analysis. Front Immunol. (2024) 15:1330386. doi: 10.3389/fimmu.2024.1330386 38495887 PMC10943692

[41] Acosta JC Banito A Wuestefeld T Georgilis A Janich P Morton JP . A complex secretory program orchestrated by the inflammasome controls paracrine senescence. Nat Cell Biol. (2013) 15:978–90. doi: 10.1038/ncb2784 23770676 PMC3732483

[42] Sturmlechner I Zhang C Sine CC van Deursen EJ Jeganathan KB Hamada N . p21 produces a bioactive secretome that places stressed cells under immunosurveillance. Sci (New York NY). (2021) 374:eabb3420. doi: 10.1126/science.abb3420 34709885 PMC8985214

[43] Hecker L Logsdon NJ Kurundkar D Kurundukar A Bernard K Hock T . Reversal of persistent fibrosis in aging by targeting Nox4-Nrf2 redox imbalance. Sci Transl Med. (2014) 6:231ra47. doi: 10.1126/scitranslmed.3008182 24718857 PMC4545252

[44] Kugel CH Douglass SM Webster MR Kaur A Liu Q Yin X . Age correlates with response to anti-PD1, reflecting age-related differences in intratumoral effector and regulatory T-cell populations. Clin Cancer Res. (2018) 24:5347–56. doi: 10.1158/1078-0432.CCR-18-1116 29898988 PMC6324578

[45] Papadopoulos D Magliozzi R Bandiera S Cimignolo I Barusolo E Probert L . Accelerated cellular senescence in progressive multiple sclerosis: A histopathological study. Ann Neurol. (2025) 97:1074–87. doi: 10.1002/ana.27195 39891488 PMC12081997

[46] Gross PS Dúran-Laforet V Ho LT Melchor GS Zia S Manavi Z . Senescent-like microglia limit remyelination through the senescence associated secretory phenotype. Nat Commun. (2025) 16:2283. doi: 10.1038/s41467-025-57632-w 40055369 PMC11889183

[47] Vladimirova O O'Connor J Cahill A Alder H Butunoi C Kalman B . Oxidative damage to DNA in plaques of MS brains. Multiple Sclerosis (Houndmills Basingstoke England). (1998) 4:413–8. doi: 10.1177/135245859800400503 9839301

[48] Dutta R McDonough J Yin X Peterson J Chang A Torres T . Mitochondrial dysfunction as a cause of axonal degeneration in multiple sclerosis patients. Ann Neurol. (2006) 59:478–89. doi: 10.1002/ana.20736 16392116

[49] Lu F Selak M O'Connor J Croul S Lorenzana C Butunoi C . Oxidative damage to mitochondrial DNA and activity of mitochondrial enzymes in chronic active lesions of multiple sclerosis. J Neurol Sci. (2000) 177:95–103. doi: 10.1016/s0022-510x(00)00343-9 10980305

[50] De Riccardis L Rizzello A Ferramosca A Urso E De Robertis F Danieli A . Bioenergetics profile of CD4(+) T cells in relapsing remitting multiple sclerosis subjects. J Biotechnol. (2015) 202:31–9. doi: 10.1016/j.jbiotec.2015.02.015 25701681

[51] Mesaros C Arora JS Wholer A Vachani A Blair IA . 8-Oxo-2'-deoxyguanosine as a biomarker of tobacco-smoking-induced oxidative stress. Free Radical Biol Med. (2012) 53:610–7. doi: 10.1016/j.freeradbiomed.2012.04.006 22613262 PMC4283839

[52] Olinski R Rozalski R Gackowski D Foksinski M Siomek A Cooke MS . Urinary measurement of 8-OxodG, 8-OxoGua, and 5HMUra: a noninvasive assessment of oxidative damage to DNA. Antioxid Redox Signaling. (2006) 8:1011–9. doi: 10.1089/ars.2006.8.1011 16771691

[53] Vasić M Topić A Marković B Milinković N Dinčić E . Oxidative stress-related risk of the multiple sclerosis development. J Med Biochem. (2023) 42:1–8. doi: 10.5937/jomb0-37546 36819128 PMC9920994

[54] Burgetova A Dusek P Uher T Vaneckova M Vejrazka M Burgetova R . Oxidative stress markers in cerebrospinal fluid of newly diagnosed multiple sclerosis patients and their link to iron deposition and atrophy. Diagn (Basel Switzerland). (2022) 12:1365. doi: 10.3390/diagnostics12061365 35741175 PMC9221788

[55] Kutzelnigg A Lucchinetti CF Stadelmann C Brück W Rauschka H Bergmann M . Cortical demyelination and diffuse white matter injury in multiple sclerosis. Brain A J Neurol. (2005) 128:2705–12. doi: 10.1093/brain/awh641 16230320

[56] Lucchinetti CF Popescu BF Bunyan RF Moll NM Roemer SF Lassmann H . Inflammatory cortical demyelination in early multiple sclerosis. N Engl J Med. (2011) 365:2188–97. doi: 10.1056/NEJMoa1100648 22150037 PMC3282172

[57] Nielsen BR Ratzer R Börnsen L von Essen MR Christensen JR Sellebjerg F . Characterization of naïve, memory and effector T cells in progressive multiple sclerosis. J Neuroimmunol. (2017) 310:17–25. doi: 10.1016/j.jneuroim.2017.06.001 28778440

[58] Naylor K Li G Vallejo AN Lee WE Koetz K Bryl E . The influence of age on T cell generation and TCR diversity. J Immunol (Baltimore Md 1950). (2005) 174:7446–52. doi: 10.4049/jimmunol.174.11.7446 15905594

[59] Cree BAC Hollenbach JA Bove R Kirkish G Sacco S Caverzasi E . Silent progression in disease activity-free relapsing multiple sclerosis. Ann Neurol. (2019) 85:653–66. doi: 10.1002/ana.25463 30851128 PMC6518998

[60] Frischer JM Bramow S Dal-Bianco A Lucchinetti CF Rauschka H Schmidbauer M . The relation between inflammation and neurodegeneration in multiple sclerosis brains. Brain. (2009) 132(5):1175–89. doi: 10.1093/brain/awp070 19339255 PMC2677799

[61] Cardenas A Ecker S Fadadu RP Huen K Orozco A McEwen LM . Epigenome-wide association study and epigenetic age acceleration associated with cigarette smoking among Costa Rican adults. Scientific Reports. (2022) 12:5628. doi: 10.1038/s41598-022-08160-w 35277542 PMC8917214

[62] Cherian AK Parikh V Wu Q Mao-Draayer Y Wang Q Blakely RD . Hemicholinium-3 sensitive choline transport in human T lymphocytes: Evidence for use as a proxy for brain choline transporter (CHT) capacity. Neurochem Int. (2017) 108:410–6. doi: 10.1016/j.neuint.2017.05.022 28577989 PMC5524217

[63] Buck MD O'Sullivan D Pearce EL . T cell metabolism drives immunity. J Exp Med. (2015) 212:1345–60. doi: 10.1084/jem.20151159 26261266 PMC4548052

[64] Martos SN Campbell MR Lozoya OA Wang X Bennett BD Thompson IJ . Single-cell analyses identify dysfunctional CD16+ CD8 T cells in smokers. Cell Rep Med. (2022) 1:100054. doi: 10.2139/ssrn.3517537 PMC7644053 33163982

[65] Fernandes JR Pinto TNC Arruda LB da Silva CB de Carvalho CR Pinto RM . Age-associated phenotypic imbalance in TCD4 and TCD8 cell subsets: comparison between healthy aged, smokers, COPD patients and young adults. Immun Ageing I A. (2022) 19:9. doi: 10.1186/s12979-022-00267-y 35164774 PMC8842531

[66] Saint-André V Charbit B Biton A Rouilly V Possémé C Bertrand A . Smoking changes adaptive immunity with persistent effects. Nature. (2024) 626:827–35. doi: 10.1038/s41586-023-06968-8 38355791 PMC10881394

[67] Tomas-Ojer P Puthenparampil M Cruciani C Docampo MJ Martin R Sospedra M . Characterization of antigen-induced CD4+ T-cell senescence in multiple sclerosis. Front Neurol. (2022) 13:790884. doi: 10.3389/fneur.2022.790884 35185762 PMC8852676

[68] Lee KA Shin KS Kim GY Song YC Bae EA Kim IK . Characterization of age-associated exhausted CD8^+^ T cells defined by increased expression of Tim-3 and PD-1. Aging Cell. (2016) 15:291–300. doi: 10.1111/acel.12435 26750587 PMC4783346

[69] Friedman GD Siegelaub AB Seltzer CC . Smoking habits and the leukocyte count. Arch Environ Health. (1973) 26:137–43. doi: 10.1080/00039896.1973.10666241 4688852

[70] Nyunoya T Monick MM Klingelhutz A Yarovinsky TO Cagley JR Hunninghake GW . Cigarette smoke induces cellular senescence. Am J Respir Cell Mol Biol. (2006) 35:681–8. doi: 10.1165/rcmb.2006-0169OC 16840774 PMC2643295

[71] Heguy A O'Connor TP Luettich K Worgall S Cieciuch A Harvey BG . Gene expression profiling of human alveolar macrophages of phenotypically normal smokers and nonsmokers reveals a previously unrecognized subset of genes modulated by cigarette smoking. J Mol Med (Berlin Germany). (2006) 84:318–28. doi: 10.1007/s00109-005-0008-2 16520944

[72] Wessler I Kilbinger H Bittinger F Unger R Kirkpatrick CJ . The non-neuronal cholinergic system in humans: expression, function and pathophysiology. Life Sci. (2003) 72:2055–61. doi: 10.1016/s0024-3205(03)00083-3 12628456

[73] Tracey KJ . The inflammatory reflex. Nature. (2002) 420:853–9. doi: 10.1038/nature01321 12490958

[74] Bertrand A Sugrue J Lou T Bourke NM Quintana-Murci L Saint-André V . Impact of socioeconomic status on healthy immune responses in humans. Immunol Cell Biol. (2024) 102:618–29. doi: 10.1111/imcb.12789 38862267

[75] Wu W Tian L Zhang W Booth JL Ainusa-Enrich E Kovats S . Long-term cigarette smoke exposure dysregulates pulmonary T cell response and IFN-γ protection to influenza virus in mouse. Respir Res. (2021) 22:112. doi: 10.1186/s12931-021-01713-z 33879121 PMC8056367

[76] Li Y Hecht SS . Carcinogenic components of tobacco and tobacco smoke: A 2022 update. Food Chem Toxicol. (2022) 165:113173. doi: 10.1016/j.fct.2022.113179 35643228 PMC9616535

[77] Long J Wang X Gao H Liu Z Liu C Miao M . Malonaldehyde acts as a mitochondrial toxin: Inhibitory effects on respiratory function and enzyme activities in isolated rat liver mitochondria. Life Sci. (2006) 79:1466–72. doi: 10.1016/j.lfs.2006.04.024 16737718

[78] Haku Y Kitaoka K Ichimaru K Hirano T Wang J Sonomura K . Active aldehydes accelerate CD8+ T cell exhaustion by metabolic alteration in the tumor microenvironment. Nat Immunol. (2026). doi: 10.1038/s41590-025-02370-w 41501155

[79] Yarosz EL Chang CH . The role of reactive oxygen species in regulating T cell-mediated immunity and disease. Immune Netw. (2018) 18:e14. doi: 10.4110/in.2018.18.e14 29503744 PMC5833121

[80] Nakahira K Hisata S Choi AM . The roles of mitochondrial damage-associated molecular patterns in diseases. Antioxid Redox Signaling. (2015) 23:1329–50. doi: 10.1089/ars.2015.6407 26067258 PMC4685486

[81] Goedtke L Sprenger H Hofmann U Schmidt FF Hammer HS Zanger UM . Polycyclic aromatic hydrocarbons activate the aryl hydrocarbon receptor and the constitutive androstane receptor to regulate xenobiotic metabolism in human liver cells. Int J Mol Sci. (2020) 22(11):372. doi: 10.3390/ijms22010372 33396476 PMC7796163

[82] Martínez-Hernández MI Acosta-Saavedra LC Hernández-Kelly LC Loaeza-Loaeza J Ortega A . Microglial activation in metal neurotoxicity: Impact in neurodegenerative diseases. BioMed Res Int. (2023) 2023(1):7389508. doi: 10.1155/2023/7389508 36760476 PMC9904912

[83] Xu H Li X Liu K Huang P Liu XJ . PM2.5 promotes macrophage-mediated inflammatory response through airway epithelial cell-derived exosomal miR-155-5p. J Inflammation Res. (2024) 17:8555–67. doi: 10.2147/JIR.S482509 39539727 PMC11559224

[84] Cao Y Sun J Wang X Zhang X Tian H Huang L . The double-edged nature of nicotine: toxicities and therapeutic potentials. Front Pharmacol. (2024) 15:1427314. doi: 10.3389/fphar.2024.1427314 39206262 PMC11350241

[85] Wu J Olsson T Hilbert J Alfredson L Hedström AK . Influence of oral tobacco versus smoking on multiple sclerosis disease activity and progression. J Neurol Neurosurg Psychiatry. (2023) 94:589–96. doi: 10.1136/jnnp-2022-330848 37001984 PMC10359558

[86] Rodgers J Friede T Vonberg FW Constantinescu CS Coles A Chataway J . The impact of smoking cessation on multiple sclerosis disease progression. Brain: A J Neurol. (2022) 145:1368–78. doi: 10.1093/brain/awab385 34623418 PMC9128822

[87] van der Vuurst de Vries RM Mescheriakova JY Runia TF Siepman TA Wokke BHA Samjin JP . Smoking at time of CIS increases the risk of clinically definite multiple sclerosis. J Neurol. (2018) 265:1010–5. doi: 10.1007/s00415-018-8780-4 29464378 PMC5937895

[88] Chatterjee S Tao JQ Johncola A Guo W Caporale A Langham MC . Acute exposure to e-cigarettes causes inflammation and pulmonary endothelial oxidative stress in nonsmoking, healthy young subjects. Am J Physiol Lung Cell Mol Physiol. (2019) 317:L155–66. doi: 10.1152/ajplung.00110.2019 31042077 PMC6734380

[89] Schoeps VA Cortese M Munger KL Mancuso JD Niebuhr DW Peng X . Smoking and multiple sclerosis risk in black people: A nested case-control study. Multiple Sclerosis Relat Disord. (2024) 82:105365. doi: 10.1016/j.msard.2023.105365 38104478 PMC10843624

[90] Lie IA Wenes K Kvistad SS Brouwer I Wergeland S Holmøy T . The effect of smoking on long-term gray matter atrophy and clinical disability in patients with relapsing-remitting multiple sclerosis. Neurol(R) Neuroimmunol Neuroinflamm. (2022) 9:e200008. doi: 10.1212/NXI.0000000000200008 35738901 PMC9223432

[91] Trapp BD Peterson J Ransohoff RM Rudick R Mörk S Bö L . Axonal transection in the lesions of multiple sclerosis. New Engl J Med. (1998) 338(5):278–85. doi: 10.1056/NEJM199801293380502 9445407

[92] Wertheimer AM Bennett MS Park B Uhrlaub JL Martinez C Pulko V . Aging and cytomegalovirus infection differentially and jointly affect distinct circulating T cell subsets in humans. J Immunol. (2014) 192(5):2143–55. doi: 10.4049/jimmunol.1301721 24501199 PMC3989163

[93] Healy BC Ali EN Guttman CRG Chitnis T Glanz BI Buckle G . Smoking and disease progression in multiple sclerosis. Archives of Neurology. (2009) 66(7):858–64. doi: 10.1001/archneurol.2009.122 19597087 PMC2754172

[94] Zivadinov R Weinstock-Guttman B Hashmi K Abdelrahman N Stosic M Dwyer M . Smoking is associated with increased lesion volumes and brain atrophy in multiple sclerosis. Neurology. (2009) 73(7):504–10. doi: 10.1212/WNL.0b013e3181b2a706 19687451 PMC2833095

[95] Tanaka E Watanabe M Fukumoto S Masaki K Yamasaki R Matsushita T . Effect of smoking on disease activity in multiple sclerosis patients treated with dimethyl fumarate or fingolimod. Multiple Sclerosis Relat Disord. (2023) 71:104513. doi: 10.1016/j.msard.2023.104513 36689892

[96] Hedström AK Olsson T Alfredsson L . Smoking is a major preventable risk factor for multiple sclerosis. Multiple Sclerosis Journal. (2016) 22(8):1021–26. doi: 10.1177/1352458515609794 26459151

[97] Scalfari A Neuhaus A Daumer M Ebers GC Muraro PA . Age and disability accumulation in multiple sclerosis. Neurology. (2011) 77:1246–52. doi: 10.1212/WNL.0b013e318230a17d 21917763 PMC3179646

[98] Fujii T Mashimo M Moriwaki Y Misawa H Ono S Horiguchi K . Physiological functions of the cholinergic system in immune cells. J Pharmacol Sci. (2017) 134:1–21. doi: 10.1016/j.jphs.2017.05.002 28552584

[99] Tremlett H Yousefi M Devonshire V Rieckmann P Zhao Y . Impact of multiple sclerosis relapses on progression diminishes with time. Neurology. (2009) 73(20):1616–23. doi: 10.1212/WNL.0b013e3181c1e44f 19890070 PMC2881858

[100] Kuhlmann T . Multiple sclerosis progression: Time for a new mechanism-driven framework. Lancet Neurol. (2017) 16(8):681–94. doi: 10.1016/S1474-4422(22)00289-7 36410373 PMC10463558

